# Steps toward Rationalization of the Enantiomeric Excess of the Sakurai–Hosomi–Denmark Allylation Catalyzed by Biisoquinoline N,N′-Dioxides Using Computations

**DOI:** 10.3390/catal11121487

**Published:** 2021-12-04

**Authors:** Pierpaolo Morgante, Coty Deluca, Tegla E. Jones, Gregory J. Aldrich, Norito Takenaka, Roberto Peverati

**Affiliations:** Chemistry Program, Florida Institute of Technology, 150 W. University Blvd., Melbourne, FL 32901, USA

**Keywords:** DFT, Lewis bases, organocatalysis, allyltrichlorosilane

## Abstract

Allylation reactions of aldehydes are chemical transformations of fundamental interest, as they give direct access to chiral homoallylic alcohols. In this work, we focus on the full computational characterization of the catalytic activity of substituted biisoquinoline-N,N′-dioxides for the allylation of 2-naphthaldehyde. We characterized the structure of all transition states as well as identified the π stacking interactions that are responsible for their relative energies. Motivated by disagreement with the experimental results, we also performed an assessment of 34 different density functional methods, with the goal of assessing DFT as a general tool for understanding this chemistry. We found that the DFT results are generally consistent as long as functionals that correctly account for dispersion interactions are used. However, agreement with the experimental results is not always guaranteed. We suggest the need for a careful synergy between computations and experiments to correctly interpret the data and use them as a design tool for new and improved asymmetric catalysts.

## 1. Introduction

**Scheme 1.**
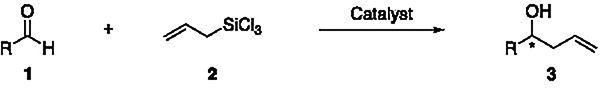
The Sakurai–Hosomi–Denmark allylation reaction.

The development of new enantioselective synthetic methods using hypervalent silicon complexes generated from chiral Lewis base catalysts and chlorosilanes is a very active and important field of research in organic synthesis and catalysis [1–3]. In particular, the Sakurai–Hosomi–Denmark allylation reaction (Scheme 1) is commonly used for the development of new chiral Lewis bases.

**Figure 1.**
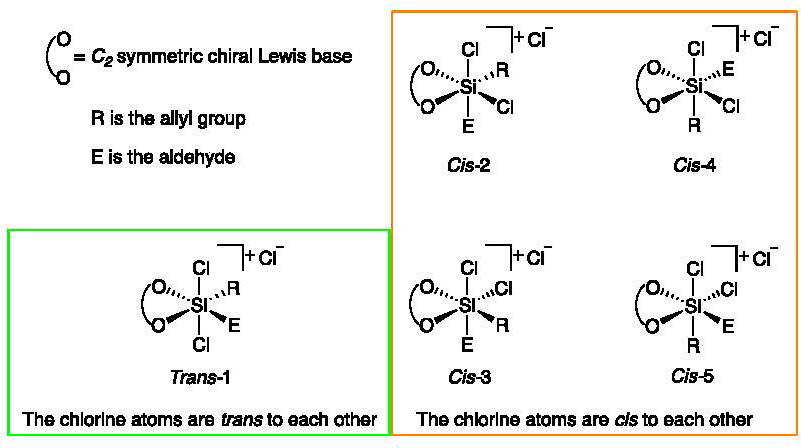
The five possible arrangements of the ligands and chlorine atoms around a hexavalent silicon atom when a *C_2_-symmetric* Lewis base (such as structures **5–9** in Figure 2) is used. See also Section S2 in the Supporting Information for a visual representation.

Significant experimental contributions in this field include the work of Denmark, Kočovský, Malkov, Nakajima, and more recently from one of us [4–11]. The stereochemical aspects of these reactions have been initially rationalized using conventional stereoelectronic arguments for the transition states (TSs) of the transient hypervalent silicon intermediates [12]. Computational work from Wheeler [13–16], has elucidated that the interaction between the ligands in these TSs play a central role for the enantioselectivity of a reaction. Specifically, a *C*_2_-*symmetric* bidentate Lewis base, RSiCl_3_, and an electrophile can produce five diastereomeric TSs with different enantioselectivities (Figure 1). The interplay between the dominating interactions in the competing TSs appears to be highly challenging to model, and questions on the possible generalization of the computational results to several substitutions of the catalysts and to different reaction substrates remain unanswered.

**Figure 2.**
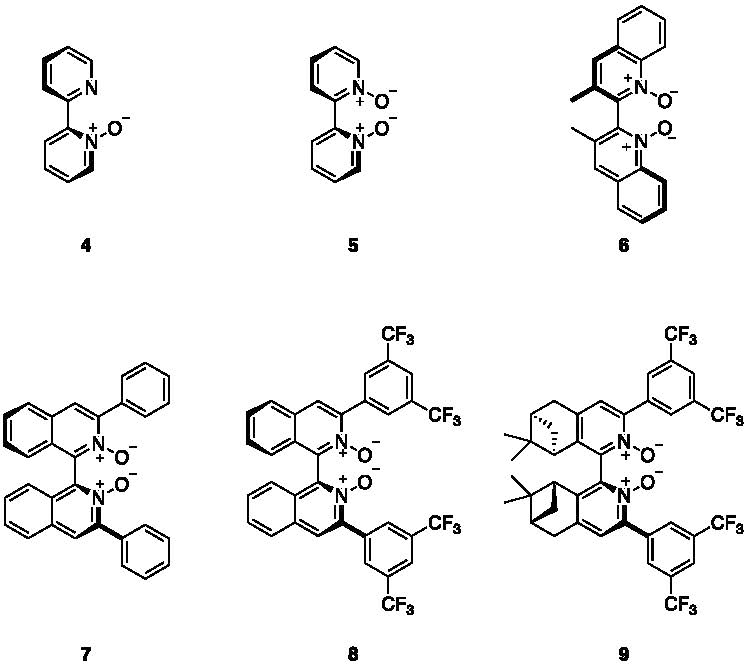
Structures of the catalysts that have been computationally characterized in the literature (**4**–**7**,**9**) as well as Takenaka’s catalyst studied in this work (**8**). Structures **5**–**9** are *C*_2_-*symmetric*; see also the label of Figure 1 and Section S2 of the Supporting Information for additional visual representations.

In 2012, Lu et al. [13] used a preliminary computational protocol to study both allylation and propargylation reactions of aromatic aldehydes using trichlorosilanes and a model bipyridine N-oxide catalyst (**4**, Figure 2), with the goal of elucidating the disparate stereoselectivities reported by Nakajima [4] for their biquinoline N,N′-dioxide catalyst (**6**). They assumed these reactions to be under Curtin–Hammett control, and they used density functional theory (DFT) calculations with the B97-D functional [17] and the TZV (2p,2d) basis set [18] to identify and characterize the relative free energies of the TSs. Their main findings were that several interactions between the ligands surrounding the silicon affect the relative ordering of the relevant transition states. They also identified a *cis*-chlorine TS to be more stable than the *trans*-chlorine one, which is in contrast to what was usually hypothesized using conventional stereoelectronic arguments at the silicon center. These results suggested that the stereoselectivity of the corresponding reactions can be modulated by changing the secondary interactions between the ligands. In a subsequent paper from the same group [14], Sepùlveda et al. expanded their previous computation to assess the performance of several DFT functionals and basis set combinations for prediction of the enantiomeric excess (*ee*) using the *C*_2_-*symmetric* catalyst **5** (Figure 1). These results confirmed that the secondary interactions between the ligands are responsible for the relative ordering of the TSs but also showed that the DFT results are in general not easily transferrable. For example, several methods that predicted an accurate *ee* for one reaction (i.e., for allylations) did not perform as well for other reactions (i.e., propargylations). Nevertheless, they confirmed the B97-D/TZV(2d,2p) as the best compromise for the prediction of experimental *ee*s. In a following article [15], they used the same protocol to perform a computational screening of several potential catalysts for the allylation and propargylation reactions of benzaldehyde, including ten variations of the biisoquinoline N,N′-dioxide catalyst, including **7**, and several others. Results of this work confirmed that the enantioselectivity of the catalyst depends on the interplay of several interactions in the arrangements of ligands. They found that π-stacking and CH/π interactions between the aldehyde and the substituents of the catalyst might further affect the relative stability of the relevant TSs and in some case even favor the *trans*-chlorine form over the *cis*-chlorine one, in contrast to their previous findings. Later work by our groups reported **8** to be the most efficient catalyst for allylation reactions [11], and preliminary DFT calculations suggested that the *trans*-chlorine form should be the preferred TS for this case. Finally, recent combined work from Wheeler’s and Malkov’s groups [16] reported experimental and computational results on the propargylation of several aldehydes using **9**. This catalyst has the same 3,5-bis (trifluoromethyl)phenyl substituents as **8**, albeit on a different scaffold. The results for this catalyst confirmed that π-stacking and CH/π interactions can substantially alter the energy landscape of the TSs. In this case, the calculations suggested that different substitutions of benzaldehyde might lead to different lowest-lying transition states and different stereoselectivities. Moreover, they also found inconsistencies between the calculated and experimental *ee*s for *o*-nitrobenzaldehyde, for which some additional stabilization due to π-stacking interactions is predicted by the calculations. This lack of consistency, united with the fact that π-stacking interactions are notoriously sensitive to substitutions, drastically limits the generalizability of these computational results from one catalyst to another. It seems evident that a detailed analysis of the structures of the TSs is required for every new synthesized catalyst.

The rationalization of the stereoselectivity of **8** was not included in Wheeler’s screening study, and because of the lack of generalization of the computational results mentioned above, we report it here for the first time. In addition, the discrepancies between some calculations and experimental results reported in [16] motivated us to conduct a larger screening of DFT methods to assess the DFT performance for the prediction of *ee*s. For several years, the accuracies of DFT exchange-correlation functionals have been notoriously inadequate for noncovalent interactions, including π-stacking. While the situation is much improved with modern meta-GGA and different flavors of dispersion-corrected functionals [19–25], it was recently reported that using different treatments of dispersion might have an effect of up to 1 kcal mol^−1^ on energy differences in the gas phase [26]. The accuracy of calculations in solution might be even lower due to the errors associated with the solvation models. Several of the competing TSs responsible for this specific chemistry are well within this difference, suggesting another potential reason for the lack of generalizability of the computational results.

## 2. Computational Methods

Each of the arrangements shown in Figure 1 can potentially yield either the R or the S enantiomer. We investigated a total of twenty transition structures, which are each labeled according to the arrangement of the chlorine atoms (either *cis* or *trans*, see the labels in Figure 1), the chair- or boat-like conformation of the six-membered ring, and the face of the aldehyde that undergoes the attach (either Re or Si, leading to the R and S enantiomers, respectively). To account for the relative position of the aldehyde and allyl group with respect to the chlorine atoms, we numbered the structures: one refers to the *trans* arrangement, while the *cis* structures are labeled from two to five. Using these conventions, the structure where the chlorine atoms are in the *trans* arrangement exposing the Re face to the attack (thus leading to the R enantiomer) and in a chair-like conformation is labeled *Trans*-1-Chair-Re. All the other labels are assigned in similar fashion.

Within the framework of density functional theory [27–29], only the most modern exchange-correlation functional approximations can describe the dispersion interactions that are responsible for the relative ordering of the TSs with sufficient accuracy. As such, we initially employed the M11 exchange-correlation functional approximation [30] because of its excellent performance for activation energies, as shown in many recent benchmark studies [21,31,32]. Given the size of the system under investigation, we used the double-ζ basis set def2-SVP [33]. All the calculations include the solvent effects of acetonitrile, using the C-PCM method [34,35], and have been performed with the Gaussian 16 program [36]. All the reported structures have been characterized as transition states with one negative frequency, and Gibbs free energies have been obtained using the harmonic approximation. Since our initial results showed differences between some of the structure of less than 2 kcal mol^−1^, we performed a benchmark of the electronic energy results with 33 additional exchange-correlation functionals, including the one identified by Wheeler et al. as the best performer for allylation and propargylation reactions (B97-D/TZV(2p,2d); see the Supporting Information for details) [13–16]. Relevant results are presented in the next section, and detailed ones for each structure are also available in the Supporting Information.

## 3. Results and Discussion

**Scheme 2.**
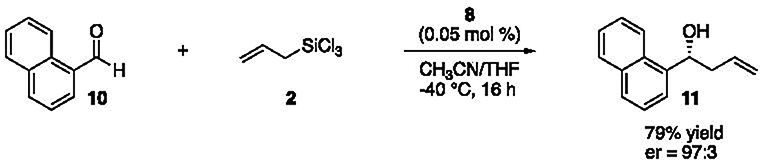
The reaction of naphtaldehyde (**10**) studied in this work. Experimental conditions are from ref. [11].

The reaction we investigated for this in silico study involves naphtaldehyde **10**, allyltrichlorosilane **2**, and catalyst **8**. The experimental conditions and the experimental enantiomeric ratio (*er*) are reported in Scheme 2 [11].

The Gibbs free energies of activation, ΔG^≠^, and the energy differences with respect to the lowest TS structure (*Trans*-1-Chair-Si), ΔΔG^≠^, are reported in Table 1 as calculated with M11/def2-SVP. In agreement with our initial intuition—motivated by our preliminary calculations—the *trans*-chlorine structures are generally lower in energy than the corresponding *cis*-chlorine ones. The *Trans*-1-Chair-Si is the lowest energy structure, which is followed by *Trans-*1-Chair-Re, *Trans*-1-Boat-Si, *Trans*-2-Chair-Re, and *Trans*-1-Boat-Re. All the remaining structures are at least 4.0 kcal mol^−1^ higher than the lowest energy one. Surprisingly enough though, these results are in disagreement with the observed experimental *ee*, which is in favor of the R isomer. Additional calculations with 34 different exchange-correlation functional approximations are reported in Table S1 in the supporting information, and they show that 29 functionals predict the *Trans-*1-Chair-Si to be lower in energy than the *Trans-*1-Chair-Re structure, which is in agreement with the M11 results. The only four functionals that predict an inverted ordering of the TSs are all non-dispersion-corrected functionals, which are not suited for studying the types of interactions present in these systems. In light of these results, we concur with Doney et al. that computationally predicted *ee*s should be within 10–20% of the experimental ones. The calculated *ee* for the allylation reaction of 2-naphthaldehyde with catalyst **8** is an unfortunate outlier to this trend.

**Figure 3.**
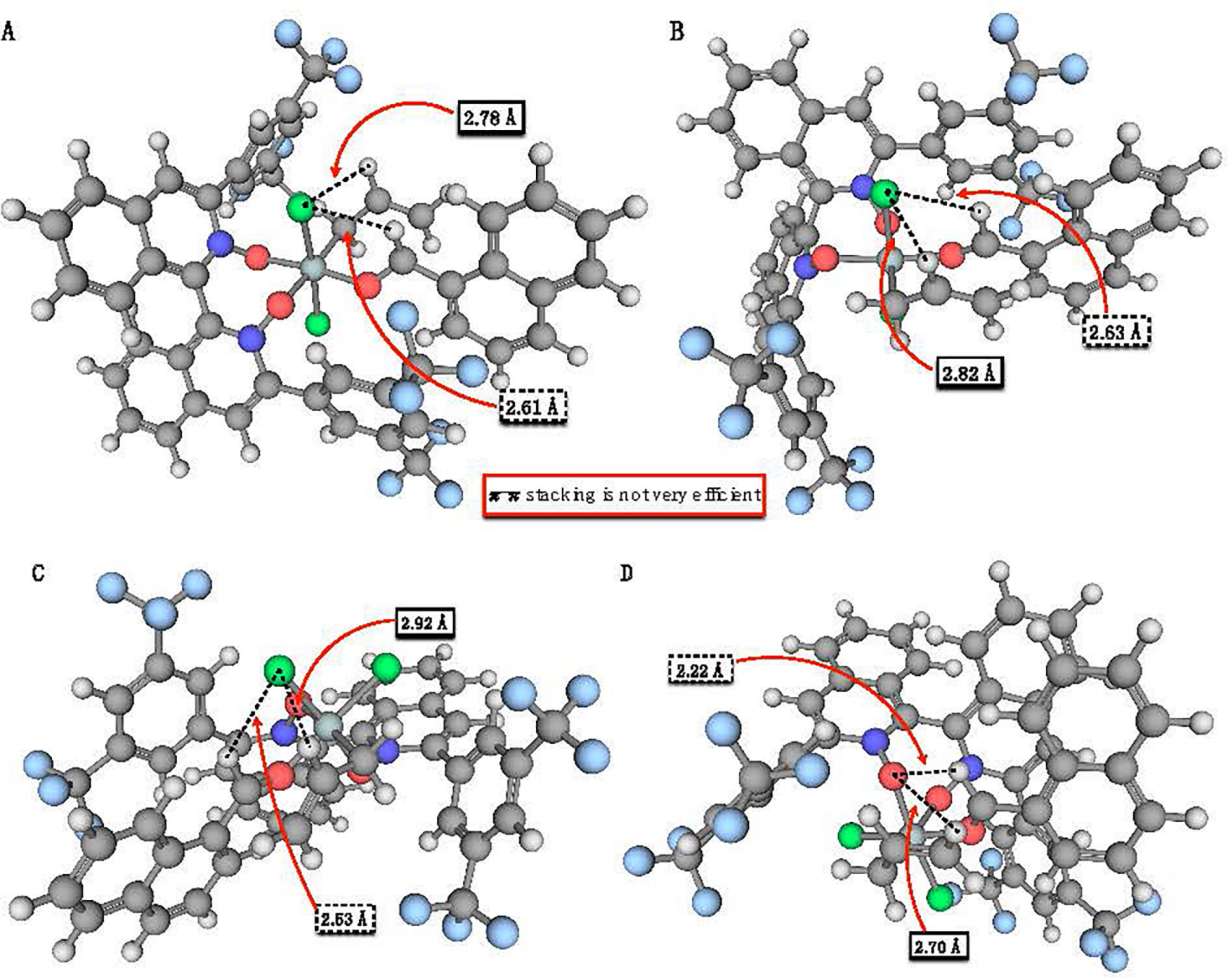
Relevant bond lengths in *Trans*-1-Chair-Re (panel **A**), *Trans*-1-Chair-Si (panel **B**), *Cis*-2-Chair-Re (panel **C**), and *Cis*-2-Chair-Si (panel **D**). The bond distances reported inside a box with a solid border refer to the allylic hydrogen, while the ones inside a box with a dotted border refer to the hydrogen of the aldehyde. The structures have been obtained at the M11/def2-SVP level of theory in acetonitrile (C-PCM).

To further understand the structural reasons behind the extra stability of the *Trans*-1- and *Cis*-2-chair structures, we turned to a visual inspection of the molecules. Wheeler et al. identified the 1,3-interactions between two C–H bonds and the chlorine atoms in the *Cis*-2 structures (labeled BP2 in their work) as the main reason behind the increased stability of the TS leading to the R enantiomer. Instead, the TS leading to the S product does not benefit from this effect. In this case, we found that the *Cis*-2-Chair-Re structure possess indeed this feature, but so do both Trans-1-Chair structures. The bond distance between the hydrogen atom of the aldehyde and the chlorine atom in the *Trans*-1-Chair-Re structure is 2.61 Å, while the distance between the hydrogen atom bound to the central carbon in the allyl group and the same chlorine is 2.78 Å (Figure 3, panel A). The distances become 2.63 Å and 2.82 Å respectively in the *Trans*-1-Chair-Si structure (Figure 3, panel B). Both cases are consistent with an appreciable 1,3-interaction, which in part explains the extra stabilization of these structures when compared to others where this interaction is absent. The distances in the *Cis*-2-Chair-Re structure are similar to those reported for the two *Trans*-1-Chair structures, being 2.53 and 2.92 Å (panel C of Figure 3). The *Cis*-2-Chair-Si structure does not present this interaction but rather a weaker interaction between the hydrogen atoms of the aldehyde and the allyl group with the oxygen atom of the Lewis base (Figure 3, panel D). We found that the *Trans*-1-Chair-Si structure is further stabilized by a π-π stacking-type interaction between the ring of the Lewis base and 2-naphthaldehyde (see Figure 3). This interaction is present exclusively in the *Trans*-1-Chair-Si transition state, and it accounts for its energy laying 1.7 kcal mol-1 below every other transition state, despite having similar 1,3-interactions between the C–H×××Cl interactions to at least two other structures, as identified above. The observation of a π stacking interaction between the substituents of the catalyst and aromatic aldehydes was also initially reported by Vaganov et al. [16] and is consistent with our findings. Perhaps not surprisingly, Vaganov et al. also report disagreement between some of their computational results and the experimental ones for these cases.

Considering the results of the DFT assessment that we reported above, the investigation of the disagreement between computation and experiment for this case and the other reported in the literature is not straightforward. Such differences might arise from the errors associated with the DFT calculation in the presence of dispersion interactions in solution. For example, we found that a difference of 1 kcal mol^−1^ affects the calculated *ee* by as much as 50% in either direction. A difference of 2 kcal mol^−1^ renders impossible the estimation of *ee* of most catalysts, since it results in changes in the calculated *ee* by as much as 80% in either direction. While they cannot be excluded a priori, such high errors for most of the available functionals are very uncommon in the DFT literature, especially because they appear for only a few specific reactions and not for other similar ones. Alternatively, possible variations in the reaction mechanisms that have not been accounted in the design of the computations are possible. For example, a reaction might not proceed under Curtin-Hammett control, it might not follow an ionic reaction mechanism, or additional solvent effects might modify the interactions in the transition states. Either way, we have to conclude that there is currently no simple computational protocol that can guarantee reliable results for all cases. Modern functionals should always be preferred, as they have been designed to make up for the deficiencies of older approximations and to have a wider range of applicability [37]. We also advise caution when interpreting the computational results, especially if they disagree with the experimental findings. The reasons behind the failure of a certain approximation are not always easy to understand, and comparison with different approximations can guide toward the choice of a better one [21,26,31,32,37]. For more complicated cases—as in this study—a comprehensive analysis including multiple functionals provides a way to validate the results, especially when the agreement with the experimental findings is questionable.

## 4. Conclusions

Our work focused on the computational study of the allylation of 2-naphthaldehyde using the bis-substituted biisoquinoline-N,N′-dioxide catalyst of Takenaka et al. (**8**). We established and validated a reliable computational protocol for the prediction of the transition states and identified the interactions that stabilize the relevant structures. Our results show that both a substantial hydrogen-chlorine 1,3-interaction and a π-π stacking interaction between the aromatic substituents of the Lewis base and the aldehyde are responsible for the extra stabilization of the *trans*-Cl configuration leading to the S isomer.

Motivated by the disagreement between the computational and experimental results, as well as similar ones reported in the literature [16], we also performed an assessment of 34 different density functional methods, with the goal of understanding the applicability of DFT as a general tool for studying this chemistry. We found that the DFT results are—in general—consistent, as long as functionals that correctly account for dispersion interactions are used. However, agreement with the experimental results is not always found for reasons that are likely not attributable to a deficiency of the DFT methods. As such, we advise caution in the interpretation of computational results. Our results question to some degree the ability to obtain computational results that are generalizable to several substitution patterns and different reactions. Modeling the relevant species is always recommended in conjunction with experimental effort for a thorough rationalization of new catalysts. We plan on expanding our study in the future to include a larger number of aldehydes and different catalysts and to eventually consider alternative reaction mechanisms.

## Supplementary Material

SI_Takenaka & Peverati_2021_Steps toward rationalization of the enantiomeric excess of the Sakurai-Hosomi-Denmark allylation catalyzed by biisofquinoline N, N'-dioxides using computations

## Figures and Tables

**Table 1.** Gibbs free energy differences calculated with respect to the reactants (second and fifth columns) and relative Gibbs free energy differences calculated with respect to the lowest-lying structure, *Trans*-1-Chair-Si (third and sixth column) for every TS involved in the reaction of allyltrichlorosilane and naphtaldehyde. All values are in kcal mol^−1^.

Structure	ΔG≠, kcal mol^−1^	ΔΔG≠,^a^ kcal mol^−1^	Structure	ΔG≠, kcal mol^−1^	ΔΔG≠,^a^ kcal mol^−1^
*Trans*-1-Boat-Si	13.0	1.78	Trans-1-Boat-Re	13.6	2.41
*Trans*-1-Chair-Si	11.2	0.00	Trans-1-Chair-Re	13.0	1.74
*Cis*-2-Boat-Si	16.3	5.03	*Cis*-2-Boat-Re	14.5	3.25
*Cis*-2-Chair-Si	15.2	3.96	*Cis*-2-Chair-Re	13.0	1.81
*Cis*-3-Boat-Si	20.4	9.18	*Cis*-3-Boat-Re	18.9	7.69
*Cis*-3-Chair-Si	17.1	5.88	*Cis*-3-Chair-Re	20.9	9.70
*Cis*-4-Boat-Si	28.6 ^b^	17.3	*Cis*-4-Boat-Re	25.6	14.4
*Cis*-4-Chair-Si	23.8	12.6	*Cis*-4-Chair-Re	21.3	10.0
*Cis*-5-Boat-Si	23.0	11.8	*Cis*-5-Boat-Re	^c^	N/A
*Cis*-5-Chair-Si	25.4	14.2	*Cis*-5-Chair-Re	19.5	8.29

a Calculated with respect to *Trans*-1-Chair-Si

b Corrected (see supporting information)

c This TS could not be located. It is expected to lie around 20.0 kcal mol^−1^.

## Data Availability

All data for this study is available within the article and the associated supplementary material, and is also available in a github repository at https://github.com/peverati/biisoquinoline_catalysts_2021.
