# Supplementary material for: Steps toward Rationalization of the Enantiomeric Excess of the Sakurai–Hosomi–Denmark Allylation Catalyzed by Biisoquinoline N,N′-Dioxides Using Computations
Source: Catalysts. Author manuscript; Available in PMC 2022 Oct 28. (PMC9615605; doi:10.3390/catal11121487)
Supplement: SI_Takenaka & Peverati_2021_Steps toward rationalization of the enantiomeric excess of the Sakurai-Hosomi-Denmark allylation catalyzed by biisofquinoline N, N'-dioxides using computations [file NIHMS1808196-supplement-1.pdf]

Supporting Information for

Steps Towards Rationalization of the Enantiomeric Excess of the Sakurai-Hosomi-Denmark Allylation Catalyzed by Biisoquinoline *N,N'*-Dioxides Using Computations.

Pierpaolo Morgante, Coty Deluca, Tegla Jones, Gregory Aldrich,

Norito Takenaka*, Roberto Peverati*

Chemistry Program, Florida Institute of Technology,

150 W. University Blvd., 32901, Melbourne (FL), United States

ntakenaka@fit.edu; rpeverati@fit.edu

Summary

Section S1: Pages S2–S4: Additional calculations on the Trans-1-Chair-Re/Si structures.

Section S2: Pages S4–8: Pictures of all the transition structures optimized with M11/def2-SVP.

Section S3: Page S8–S9: Corrections for the *Cis*-4-Boat-Si structure.

Section S4: Pages S9–S11: References.

Section S1: Additional calculations on the *Trans*-1-Chair-Re/Si structures.

The transition structures presented in **Table 1** of the main text show that the lowest energy structure is *Trans*-1-Chair-Si, the arrangement leading to the S enantiomer. This finding is in stark contrast with the experimental evidence, where the R enantiomer is obtained with an excess of 79%. This discrepancy could be due to an inherent limitation of the level of theory we chose for this study, which would invalidate its applicability altogether. To exclude this possibility, we tested thirty-two additional approximations (eight generalized gradient approximation (GGA) functionals, namely PBE,[1] PBE-D3(BJ),[1,2] BLYP,[3,4] BLYP-D3(BJ),[2–4] B97-D,[5] B97-D3(BJ),[2,5] HCTH/407,[6] HCTH/407-D3(BJ);[2,6] nine meta-GGA functionals, *i.e.* B97M-V,[7] M06-L,[8] M11-L,[9] MN15-L,[10] SCAN,[11] TPSS,[12] TPSS-D3(BJ),[2,12] τ-HCTH,[13] τ-HCTH-D3(BJ);[2,13] thirteen hybrid functionals: B3LYP,[3,4,14] B3LYP-D3(BJ),[2–4,14] PBE0,[15,16] PBE0-D3(BJ),[15,16] ωB97X-D,[17] M06-2X,[18] MN15,[19] PW6B95-D3(BJ),[2,20] τ-HCTH-hyb,[13] τ-HCTH-hyb-D3(BJ),[2,13] TPSSh,[21] TPSSh-D3(BJ),[2,21] ωB97M-V;[22] two double-hybrid functionals, B2PLYP-D3(BJ)[2,23] and DSD-PBEP86-D3(BJ))[24] with the def2-SVP basis set on the lowest-energy structures, *i.e. Trans*-1-Chair-Re and *Trans*-1-Chair-Si. We chose these approximations based on their optimal performance in recent benchmark studies,[19,25–28] with MN15,[19] ωB97M-V,[22] B2PLYP-D3(BJ)[2,23] and DSD-PBEP86-D3(BJ)[24] being among the most accurate functionals developed so far. The results of this step are collected in **Table S1**.

Table S1. Electronic energy differences (in kcal mol^{-1} and in the gas phase) between the *Trans*-1-Chair-Si and *Trans*-1-Chair-Re structures calculated with twenty-nine different functionals and the def2-SVP basis set (unless noted otherwise). The values in bold are for functionals that predict the R enantiomer to be more stable.

Functional	ΔΔE (S–R), kcal mol^{-1}	Functional	ΔΔE (S–R), kcal mol^{-1}
PBE	**–0.02**	TPSS-D3(BJ)[b]	1.14
PBE-D3(BJ)	0.84	B3LYP	0.08
BLYP	**–0.21**	B3LYP-D3(BJ)	1.31
BLYP-D3(BJ)	1.24	PBE0	0.35

B97-D	0.97	PBE0-D3(BJ)	1.17
B97-D/TZV(2p,2d)[a]	0.84	ωB97X-D	1.40
B97-D3(BJ)	1.31	M06-2X	1.72
HCTH/407	**–0.21**	M11	1.76
HCTH/407-D3(BJ)[b]	0.81	MN15	1.51
B97M-V	1.63	PW6B95-D3(BJ)	1.48
M06-L	1.61	τ-HCTHhyb	0.35
M11-L	2.28	τ-HCTHhyb-D3(BJ)[b]	1.44
MN15-L	3.12	TPSSh	0.18
SCAN	1.28	TPSSh-D3(BJ)	1.26
τ-HCTH	**–0.25**	ωB97M-V	1.67
τ-HCTH-D3(BJ)[b]	1.13	B2PLYP-D3(BJ)	1.15
TPSS	0.07	DSD-PBEP86-D3(BJ)	1.22

[a]Recommended by Wheeler and co-workers;

[b]D3(BJ) corrections obtained with a locally-modified version of the dftd3 program

Analysis of **Table S1** shows that most of the functionals, including the most accurate and reliable, agree with the results obtained at the M11/def2-SVP level of theory. Given the size of the system under investigation, it is not surprising to see that dispersion corrections must be included for a better performance. This is most striking for functionals such as PBE, BLYP, HCTH/407 and τ-HCTH, since inclusion of dispersion corrections changes the outcome from favoring the R enantiomer to favoring the S one. The effect is also prominent for the TPSS, B3LYP, PBE0, TPSSh, and τ-HCTHhyb functionals, suggesting that dispersion corrections should always be included when using these approximations, as reported in the literature.[25,26] Functionals that include dispersion interactions by construction, such as M11, MN15, and ωB97M-V, are the best option. Overall, all the functionals we tested—once they can correctly describe the system under investigation using dispersion corrections—agree with the M11/def2-SVP results. This is somewhat surprising, as many authors have reported the successful application of DFT to study the

stereochemistry of organic reactions.[29–31] The reason behind such apparent failure of all the approximations we tested is not clear, and it requires further investigation.

Section S2: Pictures of all the transition structures optimized with M11/def2-SVP.

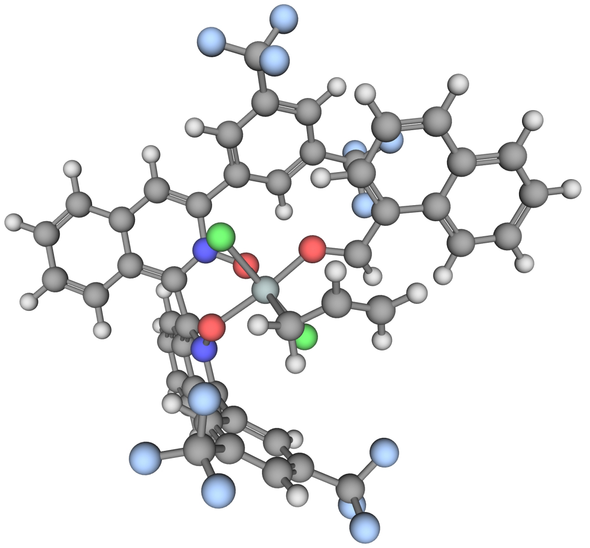

***Trans*-1-Boat-Re**

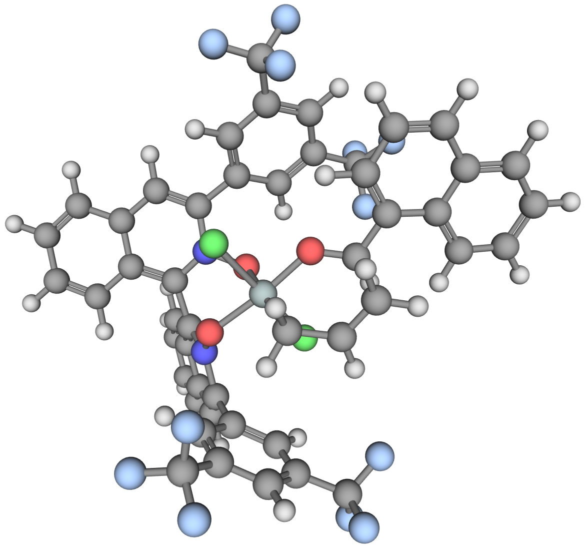

***Trans*-1-Chair-Re**

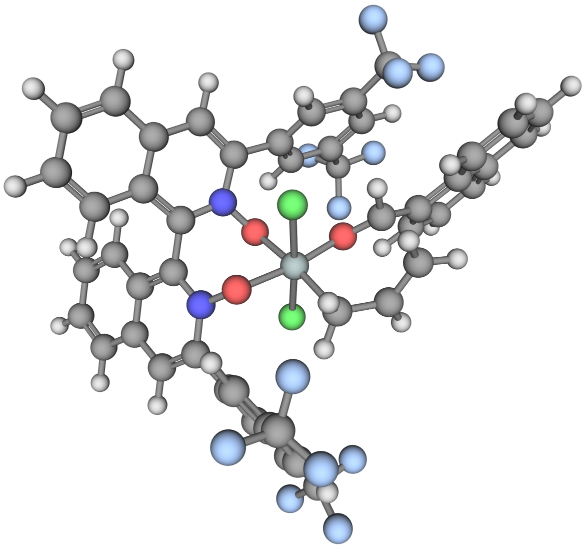

***Trans*-1-Boat-Si**

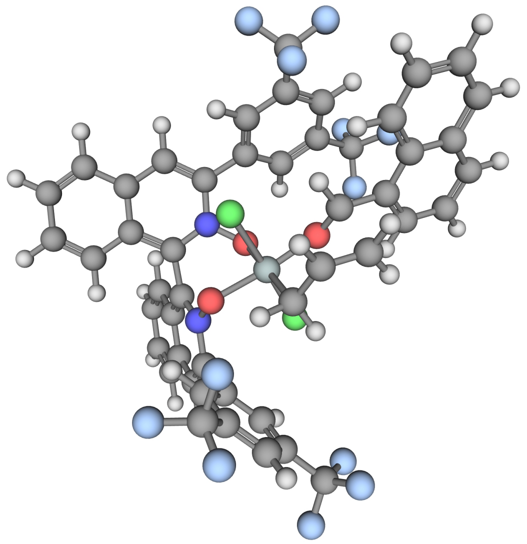

***Trans*-1-Chair-Si**

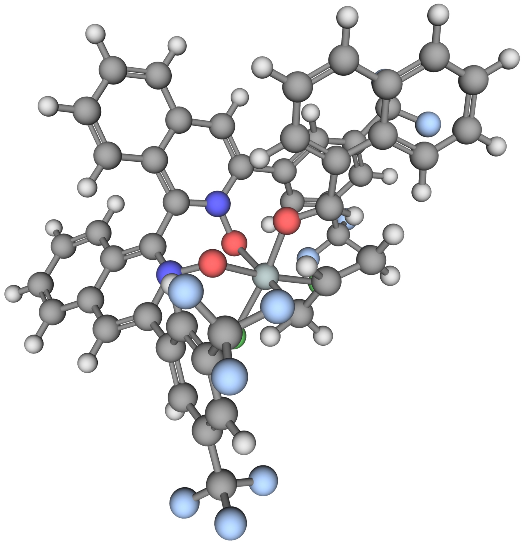

***Cis*-2-Boat-Re**

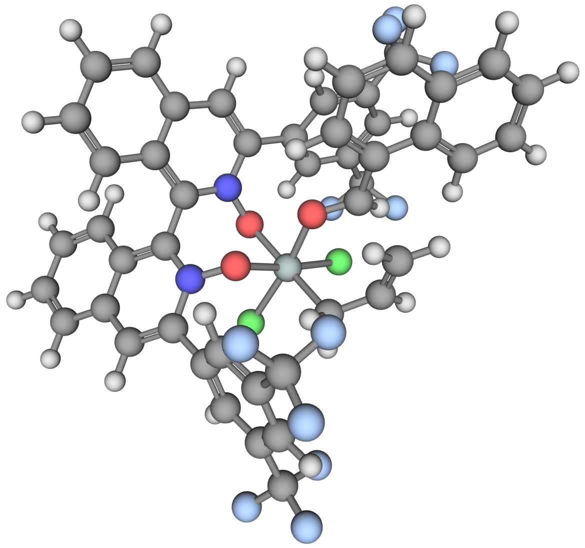

***Cis*-2-Chair-Re**

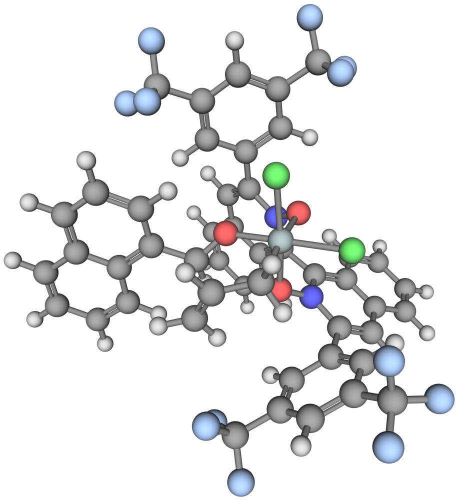

***Cis*-2-Boat-Si**

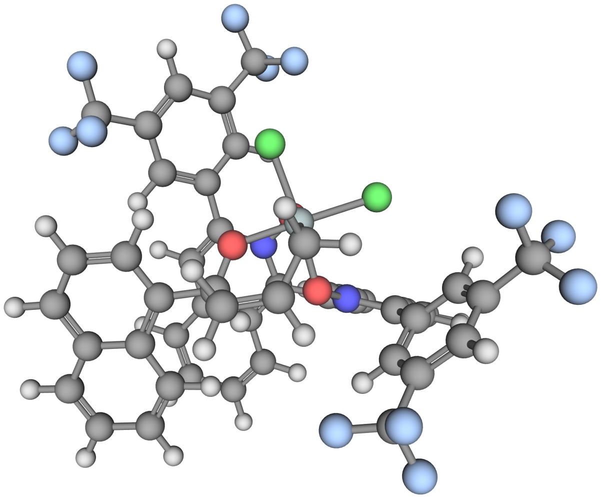

***Cis*-2-Chair-Si**

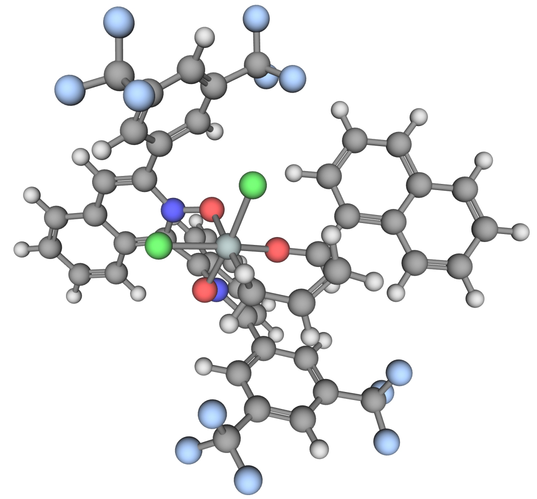

***Cis*-3-Boat-Re**

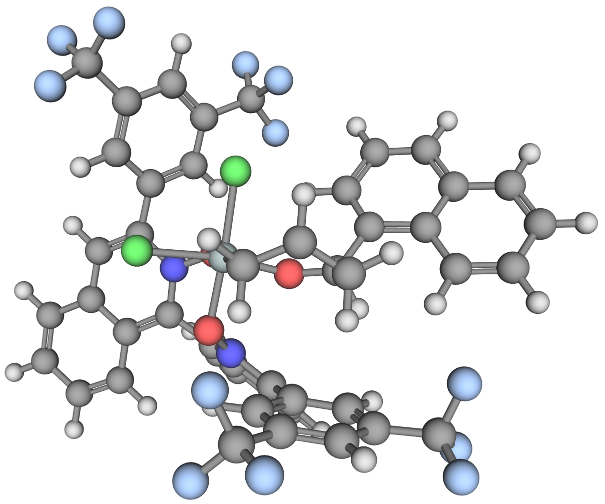

***Cis*-3-Chair-Re**

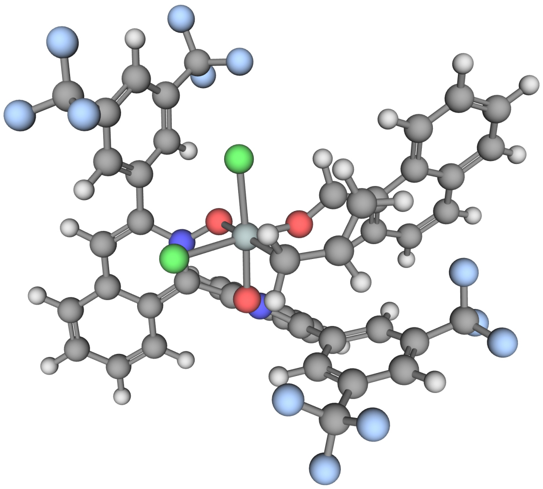

***Cis*-3-Boat-Si**

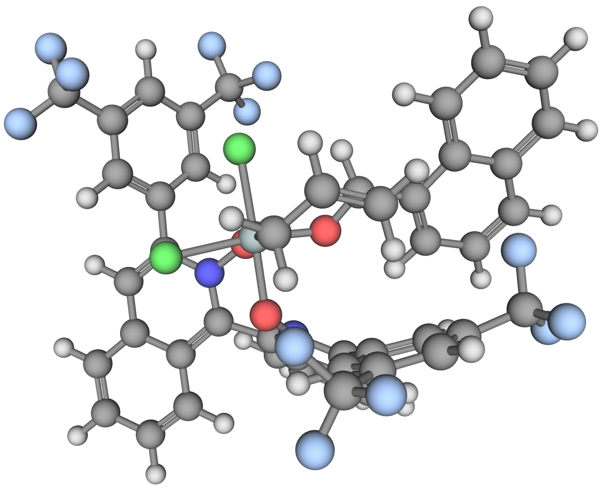

***Cis*-3-Chair-Si**

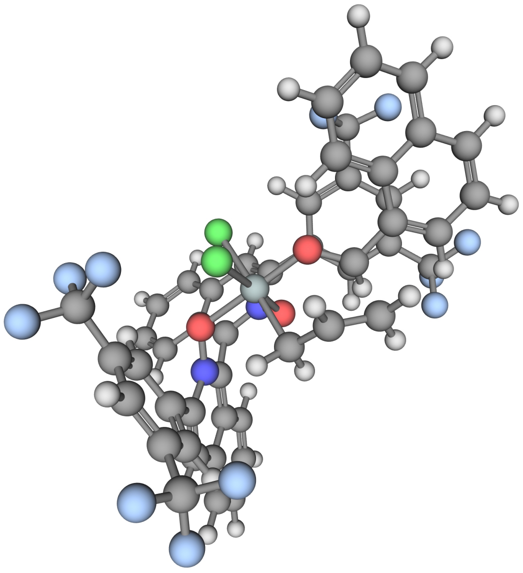

***Cis*-4-Boat-Re**

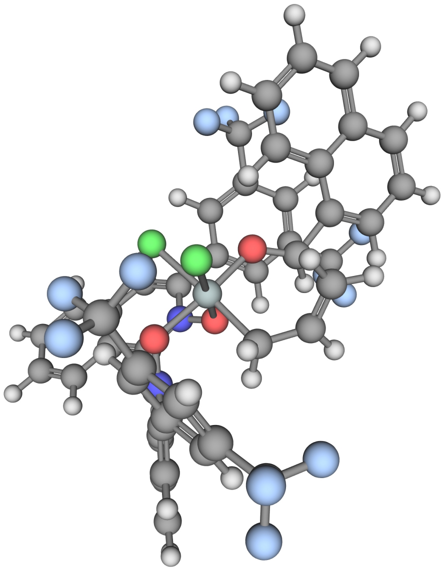

***Cis*-4-Chair-Re**

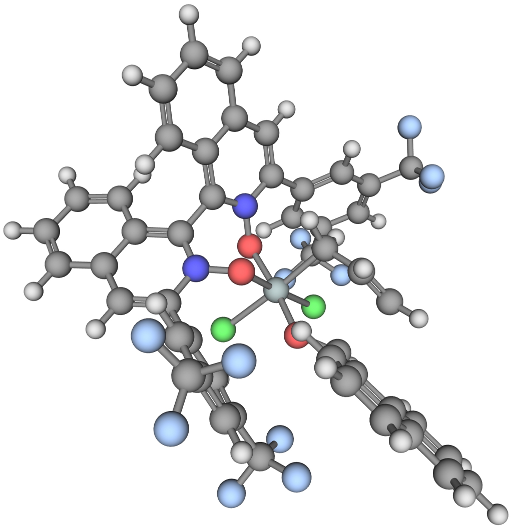

***Cis*-4-Boat-Si**

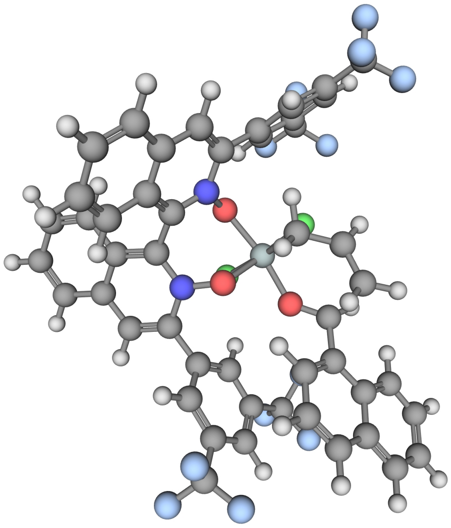

***Cis*-4-Chair-Si**

Could not be located

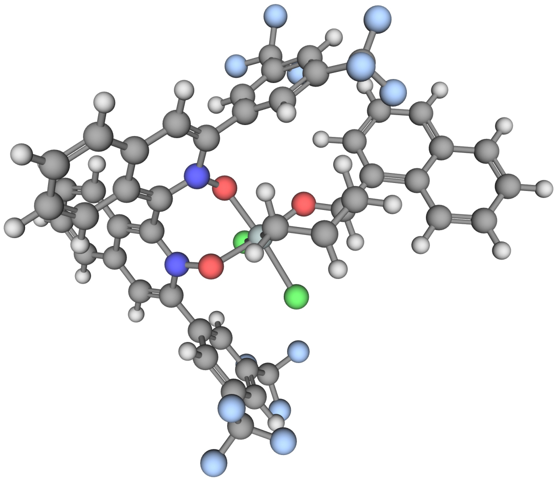

***Cis*-5-Boat-Re**

***Cis*-5-Chair-Re**

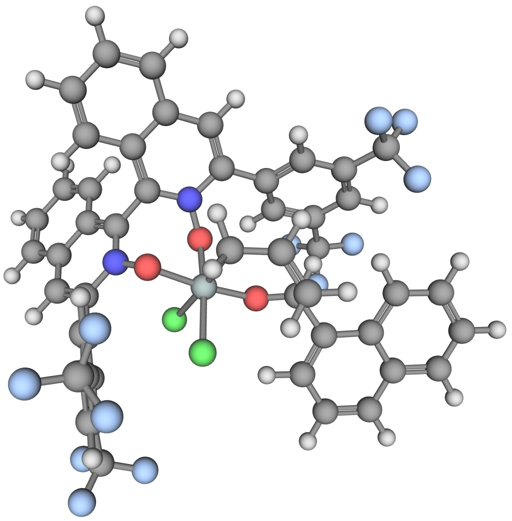

***Cis*-5-Boat-Si**

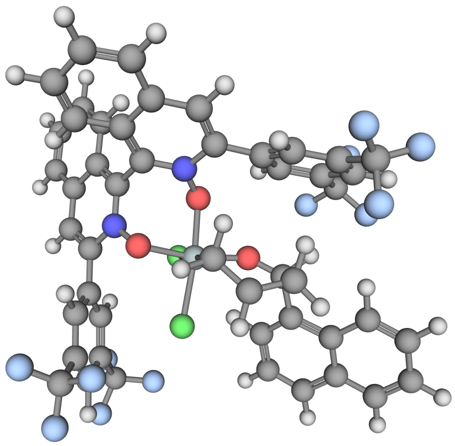

***Cis*-5-Chair-Si**

Section S3: Corrections for the *Cis*-4-Boat-Si structure.

The characterization of the *Cis*-4-Boat-Si structure revealed two negative eigenvalues of the Hessian matrix (two negative frequencies). The unwanted normal mode has a frequency of -4.25 cm^{-1}. We tried to re-optimize this transition structure using different algorithms implemented in the Gaussian

program,[32] but they were all unsuccessful. We decided to correct the values of Gibbs free energy by removing the entropy contribution due to the normal mode corresponding to the CF_3 rotation, as reported in **Table 1** of the main text.

**Molecule**	**Electronic energy, a.u.**	**Enthalpy correction, a.u.**	**Entropy, a.u.**	**Corrected entropy, XXX**	**Corrected Gibbs free energy, a.u.**
Cis-4-Boat-Si	–4585.17055721	0.738542	0.000487021	0.000476222	–4584.57400

Optimization of structure *Cis*-5-Boat-Re instead did not converge, despite multiple attempts.

Section S4: References.

(1) Perdew, J. P.; Burke, K.; Ernzerhof, M. Generalized Gradient Approximation Made Simple. *Phys. Rev. Lett.* **1996**, *77*, 3865–3868. https://doi.org/10.1103/PhysRevLett.77.3865.

(2) Grimme, S.; Ehrlich, S.; Goerigk, L. Effect of the Damping Function in Dispersion Corrected Density Functional Theory. *Journal of Computational Chemistry* **2011**, *32* (7), 1456–1465. https://doi.org/10.1002/jcc.21759.

(3) Becke, A. D. Density-Functional Exchange-Energy Approximation with Correct Asymptotic-Behavior. *Phys. Rev. A* **1988**, *38* (6), 3098–3100. https://doi.org/10.1103/PhysRevA.38.3098.

(4) Lee, C.; Yang, W.; Parr, R. G. Development of the Colle-Salvetti Correlation-Energy Formula Into a Functional of the Electron-Density. *Phys. Rev. B* **1988**, *37*, 785–789. https://doi.org/10.1103/PhysRevB.37.785.

(5) Grimme, S. Semiempirical GGA-Type Density Functional Constructed with a Long-Range Dispersion Correction. *Journal of Computational Chemistry* **2006**, *27* (15), 1787–1799. https://doi.org/10.1002/(ISSN)1096-987X.

(6) Boese, A. D.; Handy, N. C. A New Parametrization of Exchange–Correlation Generalized Gradient Approximation Functionals. *The Journal of Chemical Physics* **2001**, *114* (13), 5497–5503. https://doi.org/10.1063/1.1347371.

(7) Mardirossian, N.; Head-Gordon, M. Mapping the Genome of Meta-Generalized Gradient Approximation Density Functionals: The Search for B97M-V. *J. Chem. Phys.* **2015**, *142* (7), 074111–074132. https://doi.org/10.1063/1.4907719.

(8) Zhao, Y.; Truhlar, D. G. A New Local Density Functional for Main-Group Thermochemistry, Transition Metal Bonding, Thermochemical Kinetics, and Noncovalent Interactions. *J. Chem. Phys.* **2006**, *125* (19), 194101. https://doi.org/10.1063/1.2370993.

(9) Peverati, R.; Truhlar, D. G. M11-L: A Local Density Functional That Provides Improved Accuracy for Electronic Structure Calculations in Chemistry and Physics. *J. Phys. Chem. Lett.* **2012**, *3*, 117–124. https://doi.org/10.1021/jz201525m.

(10) Yu, H. S.; He, X.; Truhlar, D. G. MN15-L: A New Local Exchange-Correlation Functional for Kohn–Sham Density Functional Theory with Broad Accuracy for Atoms, Molecules, and Solids. *J. Chem. Theory Comput.* **2016**, *12* (3), 1280–1293. https://doi.org/10.1021/acs.jctc.5b01082.

(11) Sun, J.; Ruzsinszky, A.; Perdew, J. P. Strongly Constrained and Appropriately Normed Semilocal Density Functional. *Phys. Rev. Lett.* **2015**, *115* (3), 036402. https://doi.org/10.1103/PhysRevLett.115.036402.
(12) Tao, J.; Perdew, J. P.; Staroverov, V. N.; Scuseria, G. E. Climbing the Density Functional Ladder: Nonempirical Meta–Generalized Gradient Approximation Designed for Molecules and Solids. *Phys. Rev. Lett.* **2003**, *91* (14), 146401. https://doi.org/10.1103/PhysRevLett.91.146401.
(13) Boese, A. D.; Handy, N. C. New Exchange-Correlation Density Functionals: The Role of the Kinetic-Energy Density. *J. Chem. Phys.* **2002**, *116* (22), 9559–9569. https://doi.org/10.1063/1.1476309.
(14) Becke, A. D. Density-functional Thermochemistry. III. The Role of Exact Exchange. *J. Chem. Phys.* **1993**, *98* (7), 5648–5652. https://doi.org/10.1063/1.464913.
(15) Adamo, C.; Barone, V. Toward Reliable Density Functional Methods without Adjustable Parameters: The PBE0 Model. *J. Chem. Phys.* **1999**, *110* (13), 6158–6170. https://doi.org/10.1063/1.478522.
(16) Ernzerhof, M.; Scuseria, G. E. Assessment of the Perdew–Burke–Ernzerhof Exchange-Correlation Functional. *J. Chem. Phys.* **1999**, *110* (11), 5029–5036. https://doi.org/10.1063/1.478401.
(17) Chai, J.-D.; Head-Gordon, M. Long-Range Corrected Hybrid Density Functionals with Damped Atom–Atom Dispersion Corrections. *Phys. Chem. Chem. Phys.* **2008**, *10* (44), 6615. https://doi.org/10.1039/b810189b.
(18) Zhao, Y.; Truhlar, D. G. The M06 Suite of Density Functionals for Main Group Thermochemistry, Thermochemical Kinetics, Noncovalent Interactions, Excited States, and Transition Elements: Two New Functionals and Systematic Testing of Four M06-Class Functionals and 12 Other Functionals. *Theor Chem Acc* **2008**, *120* (1–3), 215–241. https://doi.org/10.1007/s00214-007-0310-x.
(19) Yu, H. S.; He, X.; Li, S. L.; Truhlar, D. G. MN15: A Kohn–Sham Global-Hybrid Exchange–Correlation Density Functional with Broad Accuracy for Multi-Reference and Single-Reference Systems and Noncovalent Interactions. *Chem. Sci.* **2016**, *7* (8), 5032–5051. https://doi.org/10.1039/C6SC00705H.
(20) Zhao, Y.; Truhlar, D. G. Design of Density Functionals That Are Broadly Accurate for Thermochemistry, Thermochemical Kinetics, and Nonbonded Interactions. *J. Phys. Chem. A* **2005**, *109* (25), 5656–5667. https://doi.org/10.1021/jp050536c.
(21) Staroverov, V. N.; Scuseria, G. E.; Tao, J.; Perdew, J. P. Comparative Assessment of a New Nonempirical Density Functional: Molecules and Hydrogen-Bonded Complexes. *J. Chem. Phys.* **2003**, *119* (23), 12129–12137. https://doi.org/10.1063/1.1626543.
(22) Mardirossian, N.; Head-Gordon, M. *ω* B97M-V: A Combinatorially Optimized, Range-Separated Hybrid, Meta-GGA Density Functional with VV10 Nonlocal Correlation. *J. Chem. Phys.* **2016**, *144* (21), 214110. https://doi.org/10.1063/1.4952647.
(23) Grimme, S. Semiempirical Hybrid Density Functional with Perturbative Second-Order Correlation. *J. Chem. Phys.* **2006**, *124* (3), 034108. https://doi.org/10.1063/1.2148954.
(24) Kozuch, S.; Martin, J. M. L. DSD-PBEP86: In Search of the Best Double-Hybrid DFT with Spin-Component Scaled MP2 and Dispersion Corrections. *Phys. Chem. Chem. Phys.* **2011**, *13* (45), 20104. https://doi.org/10.1039/c1cp22592h.
(25) Mardirossian, N.; Head-Gordon, M. Thirty Years of Density Functional Theory in Computational Chemistry: An Overview and Extensive Assessment of 200 Density Functionals. *Mol. Phys.* **2017**, *115* (19), 2315–2372. https://doi.org/10.1080/00268976.2017.1333644.
(26) Goerigk, L.; Hansen, A.; Bauer, C.; Ehrlich, S.; Najibi, A.; Grimme, S. A Look at the Density Functional Theory Zoo with the Advanced GMTKN55 Database for General Main Group Thermochemistry, Kinetics and Noncovalent Interactions. *Physical Chemistry Chemical Physics* **2017**, *19* (48), 32184–32215. https://doi.org/10.1039/C7CP04913G.
(27) Najibi, A.; Goerigk, L. The Nonlocal Kernel in van Der Waals Density Functionals as an Additive Correction: An Extensive Analysis with Special Emphasis on the B97M-V and $ømega$B97M-V

Approaches. *J. Chem. Theory Comput.* **2018**, *14* (11), 5725–5738. https://doi.org/10.1021/acs.jctc.8b00842.

(28) Mehta, N.; Casanova-Páez, M.; Goerigk, L. Semi-Empirical or Non-Empirical Double-Hybrid Density Functionals: Which Are More Robust? *Phys. Chem. Chem. Phys.* **2018**, *20* (36), 23175–23194. https://doi.org/10.1039/C8CP03852J.

(29) Lu, T.; Porterfield, M. A.; Wheeler, S. E. Explaining the Disparate Stereoselectivities of N-Oxide Catalyzed Allylations and Propargylations of Aldehydes. *Org. Lett.* **2012**, *14* (20), 5310–5313. https://doi.org/10.1021/ol302493d.

(30) Sepúlveda, D.; Lu, T.; Wheeler, S. E. Performance of DFT Methods and Origin of Stereoselectivity in Bipyridine N,N′-Dioxide Catalyzed Allylation and Propargylation Reactions. *Org. Biomol. Chem.* **2014**, *12* (41), 8346–8353. https://doi.org/10.1039/C4OB01719F.

(31) Barbero, M.; Cadamuro, S.; Dughera, S.; Ghigo, G.; Marabello, D.; Morgante, P. Efficient Alkylation of Cyclic Silyl Enol Ethers by Diarylmethylium Salts. *Tetrahedron Lett.* **2016**, *57* (42), 4758–4762. https://doi.org/10.1016/j.tetlet.2016.09.042.

(32) Frisch, M. J.; Trucks, G. W.; Schlegel, H. B.; Scuseria, G. E.; Robb, M. A.; Cheeseman, J. R.; Scalmani, G.; Barone, V.; Petersson, G. A.; Nakatsuji, H.; Li, X.; Caricato, M.; Marenich, A. V.; Bloino, J.; Janesko, B. G.; Gomperts, R.; Mennucci, B.; Hratchian, H. P.; Ortiz, J. V.; Izmaylov, A. F.; Sonnenberg, J. L.; Williams-Young, D.; Ding, F.; Lipparini, F.; Egidi, F.; Goings, J.; Peng, B.; Petrone, A.; Henderson, T.; Ranasinghe, D.; Zakrzewski, V. G.; Gao, J.; Rega, N.; Zheng, G.; Liang, W.; Hada, M.; Ehara, M.; Toyota, K.; Fukuda, R.; Hasegawa, J.; Ishida, M.; Nakajima, T.; Honda, Y.; Kitao, O.; Nakai, H.; Vreven, T.; Throssell, K.; Montgomery, J. A., Jr.; Peralta, J. E.; Ogliaro, F.; Bearpark, M. J.; Heyd, J. J.; Brothers, E. N.; Kudin, K. N.; Staroverov, V. N.; Keith, T. A.; Kobayashi, R.; Normand, J.; Raghavachari, K.; Rendell, A. P.; Burant, J. C.; Iyengar, S. S.; Tomasi, J.; Cossi, M.; Millam, J. M.; Klene, M.; Adamo, C.; Cammi, R.; Ochterski, J. W.; Martin, R. L.; Morokuma, K.; Farkas, O.; Foresman, J. B.; Fox, D. J. *Gaussian 16 Revision A.03*; Gaussian, Inc.: Wallingford, CT, 2016.
